# Supplementary material for: Identification of photoperiod-induced specific miRNAs in the adrenal glands of Sunite sheep (Ovis aries)
Source: Front Vet Sci. 2022 Jul 22;9:888207. doi: 10.3389/fvets.2022.888207 (PMC9354845; doi:10.3389/fvets.2022.888207)
Supplement: Supplementary file 1 [file Data_Sheet_1.ZIP › Supplementary file/miRDeep2 file.zip!/miRDeep2 file/Novel_Structure/SPLP42A1/Novel_530.pdf]

Provisional ID	:	NC_019466.2_14251
Score total	:	63.4
Score for star read(s)	:	3.9
Score for read counts	:	56.2
Score for mfe	:	1.7
Score for randfold	:	1.6
Score for cons. seed	:	
Total read count	:	122
Mature read count	:	110
Loop read count	:	0
Star read count	:	12

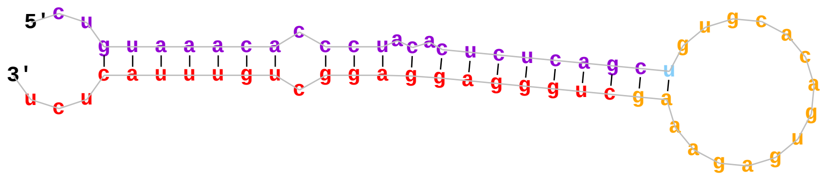

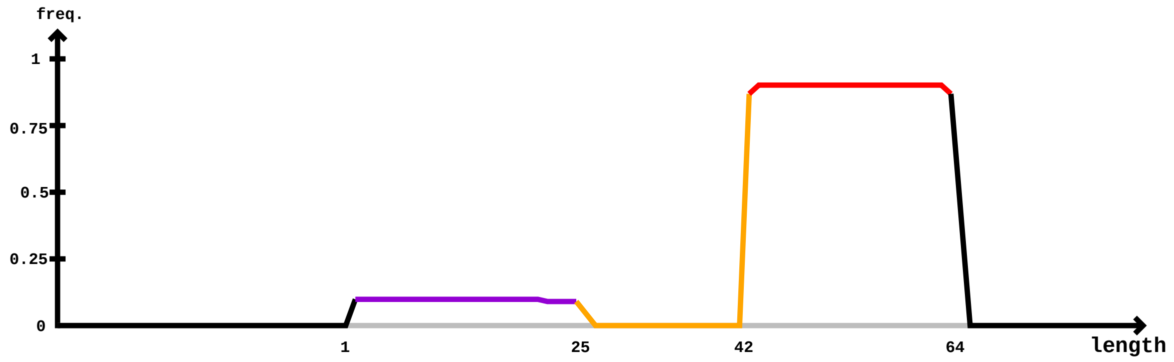

Star Mature

```
5'- ccgccccagagagcacugagggccagauacuguaaacacccuacacucucagcugugcacagugagaaagcugggaggaggcuguuuacucucucugccuuggaagucaac   -3'    obs
    ccgccccagagagcacugagggccagauacuguaaacacccuacacucucagcugugcacagugagaaagcugggaggaggcuguuuacucucucugccuuggaagucaac          exp
    ..(((((((......))).))))((((....(((((((.(((.(.(((((((((..............))))))))))))).)))))))....))))..............   reads    mm    sample
    .............................cuguaaacacccuacacucu..............................................................   1        0     seq
    .............................cuguaaacacccuacacucucagc..........................................................   7        0     seq
    .............................cuguaaacacccuacacucucagcu.........................................................   4        0     seq
    ......................................................................cugggaggaggcuguuuacuc....................   4        0     seq
    ......................................................................cugggaggaggcuguuuacucu...................   99       0     seq
    ......................................................................cugggaggaggcuguuuacucuc..................   3        0     seq
    .......................................................................ugggaggaggcuguuuacucu...................   3        0     seq
    .......................................................................ugggaggaggcuguuuacucuc..................   1        0     seq
```
